# Deep learning for automatic segmentation of thigh and leg muscles

**DOI:** 10.1007/s10334-021-00967-4

**Published:** 2021-10-19

**Authors:** Abramo Agosti, Enea Shaqiri, Matteo Paoletti, Francesca Solazzo, Niels Bergsland, Giulia Colelli, Giovanni Savini, Shaun I. Muzic, Francesco Santini, Xeni Deligianni, Luca Diamanti, Mauro Monforte, Giorgio Tasca, Enzo Ricci, Stefano Bastianello, Anna Pichiecchio

**Affiliations:** 1 Advanced Imaging and Radiomics Center, Neuroradiology Department, IRCCS Mondino Foundation, Pavia, Italy; 2 Dipartimento di Matematica, Università degli Studi di Pavia, Pavia, Italy; 3 School of Specialization in Clinical Pharmacology and Toxicology Center of research in Medical Pharmacology, School of medicine University of Insubria, Varese, Italy; 4 Buffalo Neuroimaging Analysis Center, Department of Neurology, Jacobs School of Medicine and Biomedical Sciences and University of Buffalo, The State University of New York, Buffalo, NY USA; 5 INFN, Pavia Group, Pavia, Italy; 6 Department of Neuroradiology, IRCCS Humanitas Research Hospital, Milano, Italy; 7 University of Pavia, Pavia, Italy; 8 Division of Radiological Physics, Department of Radiology, University Hospital Basel, Basel, Switzerland; 9 Department of Biomedical Engineering, University of Basel, Allschwil, Basel, Switzerland; 10 Neuro-oncology Unit, IRCCS Mondino Foundation, Pavia, Italy; 11 Unitá Operativa Complessa di Neurologia, Fondazione Policlinico Universitario A. Gemelli IRCCS, Roma, Italy; 12 Department of Brain and Behavioural Sciences, University of Pavia, Pavia, Italy

**Keywords:** Deep learning, Muscle segmentation, Magnetic resonance imaging

## Abstract

**Objective:**

In this study we address the automatic segmentation of selected muscles of the thigh and leg through a supervised deep learning approach.

**Material and methods:**

The application of quantitative imaging in neuromuscular diseases requires the availability of regions of interest (ROI) drawn on muscles to extract quantitative parameters. Up to now, manual drawing of ROIs has been considered the gold standard in clinical studies, with no clear and universally accepted standardized procedure for segmentation. Several automatic methods, based mainly on machine learning and deep learning algorithms, have recently been proposed to discriminate between skeletal muscle, bone, subcutaneous and intermuscular adipose tissue. We develop a supervised deep learning approach based on a unified framework for ROI segmentation.

**Results:**

The proposed network generates segmentation maps with high accuracy, consisting in Dice Scores ranging from 0.89 to 0.95, with respect to “ground truth” manually segmented labelled images, also showing high average performance in both mild and severe cases of disease involvement (i.e. entity of fatty replacement).

**Discussion:**

The presented results are promising and potentially translatable to different skeletal muscle groups and other MRI sequences with different contrast and resolution.

## Introduction

Recent technical advances of muscle MRI imaging have led to an evolution from traditional qualitative evaluation into what is currently known as quantitative imaging (qMRI), in which a large amount of diagnostically relevant information (such as fat substitution and edema) can be quantified and extracted from muscles of subjects affected by neuromuscular diseases [7, 23, 28]. By using quantitative indicators, it is possible to make objective comparisons across subjects or time points to evaluate the natural history of disease progression or to use those parameters as potential outcome measures of therapeutic approaches. Muscle imaging protocols in the setting of qMRI often include several quantitative sequences, with the aim of evaluating different parameters, mainly intramuscular fat component (fat fraction, FF) and intramuscular free water relaxation (water T2, w-T2), but also diffusivity properties, size (muscle volume, cross-sectional area, CSA) etc. To extract quantitative data, drawing precise regions of interest (ROI) on selected muscles is crucial. The acquisition of multiple sequences on the same region also potentially requires registering ROIs to different datasets; such a process adds the further task to manually correct the registered ROIs in the final space where data are eventually extracted for statistical analysis.

Up to now, manual drawing of ROIs has been considered the gold standard for the extraction of quantitative data from muscles in clinical studies [5, 24]. It requires dedicated and experienced human operators, long processing times and training curves, but also the necessity to select certain volumes of the entire muscle to limit the operator workload. Although muscle segmentation algorithms are not a novel concept (e.g. [6]), recent advances in hardware (offering faster processing) and in software/algorithms (new neural networks) made the potential much more promising. Therefore, the application of automatic tools to this field, mainly based on machine learning techniques and deep neural networks, already appears as particularly promising with the aim to accelerate data extraction and analysis and eventually go beyond the manual process of ROI drawing and correction. A complete overview of the evolution of the MR image segmentation strategies is reported in [21]. Indeed, up to now automated segmentation tools have been successfully used to discriminate thigh tissues into skeletal muscle, bone, subcutaneous adipose tissue and intermuscular adipose tissue. In particular, recent studies applied diverse approaches including variational segmentation methods combined with statistical clustering–based techniques on T1-weighted scans [10, 22], machine-learning classification techniques on intensity-based features extracted from multi-contrast Dixon scans [29], Deep Neural Networks (DNN) methods based on convolutional architectures combined with variational contour detector on T1-w scans [30] and DNN methods based on an encoder–decoder U-net architecture [27] combined with a clustering algorithm on T2 and proton density (PD) maps from multi spin echo scans [3]. Finally, Anwar et al. applied a semi-supervised deep learning approach based on an encoder–decoder architecture on multi-contrast Dixon scans [4]. This latter work provided a unified framework to automatically segment both the multiple tissues regions and the edges of the fascia lata, which separates the adipose tissue domain into subcutaneous and inter-muscular.

All these aforementioned methods provided a high level of accuracy of the generated segmentation maps with respect to ground truth labelled images, ranging from 0.8 to 0.97 values of Dice Similarity Coefficient (DSC, a representative metrics of similarity between the segmented and ground truth maps) for the different tissues, with the deep learning-based methods performing better in the cases of severe fat substitution [3, 10]. Indeed, Gadermayr et al. showed that classical variational and machine learning segmentation methods worked well mainly in mildly involved subjects (i.e. with a low degree of fat replacement of muscular tissue), but actually had lower accuracy when examining subjects with advanced disease where fat replacement was predominant [10]. In particular, they obtained average levels of DSC accuracies of 0.90–0.95 for tissue segmentation in mild and moderate cases, whereas they obtained average DSC values of 0.67–0.85 in severely involved cases. The application of DNN methods in discriminating muscle tissues yielded to higher performances for severe cases. Other authors, in fact, found average DSC values of 0.93–0.96, depending on the input data type of the networks [3, 30].

As for the automatic segmentation of individual muscle regions, atlas-based approaches have been proposed in [16] for the automatic segmentation of four muscles of the quadriceps femoris from T1-weighted scans of healthy subjects. In the latter work different registration methods, guided by an initial discrimination of thigh tissues obtained by means of a clustering algorithm, were evaluated, obtaining average DSCs ranging from 0.72 to 0.94 for the different muscles. Recently, Ding et al. reported a deep learning approach based on the U-net architecture which was applied to automatically segment 4 functional muscle groups of the thigh from multi–contrast Dixon scans, obtaining an average DSC on the training dataset $>0.85$ [9]. The obtained DNN-generated segmentations were shown to be unsuitable for patients with markedly severe fat infiltration, since limited data of such cases were available to train their network. Indeed, they found average DSC values of 0.85–0.93 for the single thigh muscles considered, with the lowest value corresponding to the smallest muscle, but they declared (without further investigations) that their DNN was not suitable for patients with severe fat infiltration [9].

Moreover, in [26] a cascade 3-D convolutional DNN segmentation framework, consisting of two-stage process, was designed to capture location and detailed features of muscles, reporting DSCs values of 0.78–0.97 for small and large muscles, respectively.

In the present work, as a further step towards the automatization of muscle ROI drawing, we aimed to develop an automatic segmentation tool based on deep learning techniques to create single-muscle segmentation maps at thigh and leg level, starting from manually segmented multi-contrast quantitative muscle MRI scans of both healthy subjects and patients affected by two different neuromuscular diseases. In the interest of reproducibility and of benefiting the community, we are sharing the resulting automatic segmentation tool as an open-source repository, available at [2].

## Materials and methods

### Subjects

For this project, we included 54 subjects (6 healthy controls and 48 patients affected by facioscapulohumeral dystrophy (FSHD) ($n=30$) and by amyotrophic lateral sclerosis (ALS) ($n=18$), that presented muscle alterations. Each subject was scanned at different time points (up to three). Subjects gave their informed consent to the examination. This study was approved by the Local Ethics Committee.

### MRI acquisition

All examinations were performed on a 3T MRI whole-body scanner (Skyra, Siemens Healthineers AG Erlangen, Germany) using integrated spine and body surface coils. The patient was lying supine in the scanner with 18–channel phased–array coils positioned either on the thighs and the legs during acquisition, with simultaneous acquisition of both sides (total scanning time of approximately 20 min for the thighs and 15 min for legs). The MRI protocol included a 3D six-point multi-echo gradient echo (GRE) sequence with interleaved echo sampling (matrix size = $432\times 432\times 52$ for the thighs, $432\times 432\times 36$ for the legs, TR = 35 ms, TE = 1.7–9.2 ms, resolution = $1.04\times 1.04\times 5.0 \text { mm}^3$, bandwidth 1050 Hz/Px, flip angle $7^\circ$) and a 2D multi-slice multi-echo spin echo (MESE) sequence (matrix size = $384\times 192\times 7$ for the thighs, $384\times 192\times 5$ for the legs, TE = 10.9 ms both for the first TE and the echo spacing, TR = 4100.0 ms, resolution = $1.2\times 1.2\times 10.0 mm^3$, slice gap=30 mm, 17 echo times) at thigh and leg level.

### Post-processing of MRI sequences

A total of 12 muscle ROIs per thigh and 6 muscle ROIs per leg for each side were manually drawn by a single experienced operator using ITK-snap v3.0 [31]. ROIs were drawn on the first echo images of the MESE sequence by an expert operator (FS) with 3 years of experience, avoiding the muscle fascia and bone contours of the femur and tibia.

For what concerns the thigh, ROIs were drawn in the inner thigh slices (5 out of 7) of the MESE acquisition, equidistant from the femur head and the tip of the patella, and were subsequently registered to the multi-echo GRE dataset with the creation of new corresponding ROIs, which were manually adjusted by the same operator. Two additional ROIs were drawn in the GRE space in the neighboring slices to the medial registered slice, ending in a final number of 7 slices per thigh segmented.

For what concerns the leg, segmentation was performed in the third slice of the MESE acquisition and then subsequently registered to the multi-echo GRE dataset where it was manually adjusted. Two additional ROIs were drawn in the multi-echo GRE dataset on the neighboring slices, ending in a final number of 3 slices per leg segmented.

The slices to be segmented were chosen as the most representative of the upper, middle and lower thigh, and, for simplicity, only for the middle portion of the lower leg (also to include all the most important muscles that may not be represented especially in the lower slices closer to the ankle).

### Training, validation and test datasets

We separated the available dataset of scanned subjects into training and validation subsets, for the DNN learning process, and a test subset for its testing. 44 subjects (comprising the 6 healthy controls) at the different time points, for a total number of 110 scans, were included in the training and validation subsets, whereas remaining 10 patients at their initial scan time-point were included in the test subset.

A total number of 770 thigh and 330 leg slices with corresponding manually-drawn ground truth segmentations were thus available as a working dataset for the DNN learning process and cross-validation.

### Preprocessing and data augmentation

**Fig. 1:**
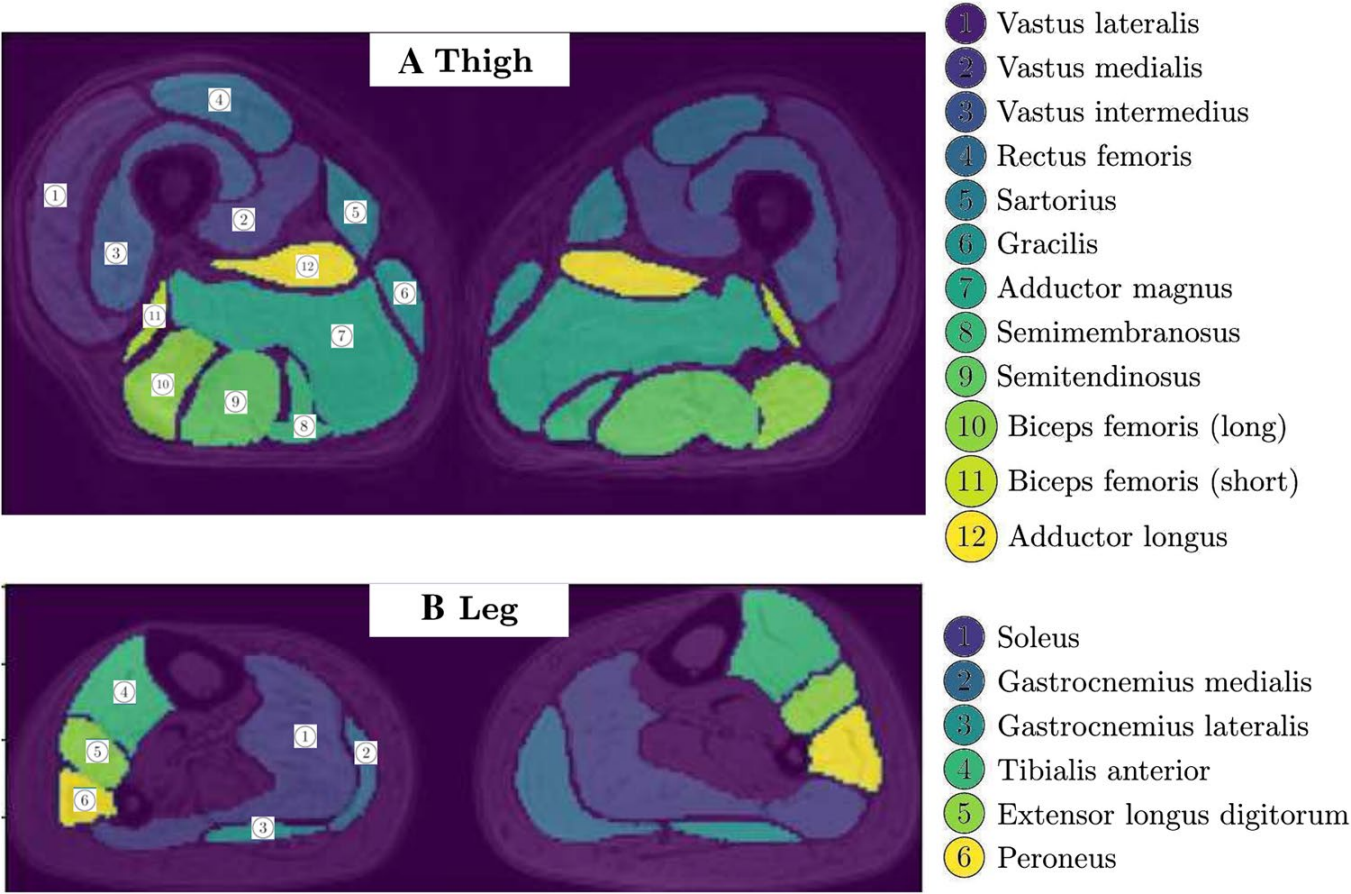
Illustrative example of thigh and leg slices from MRI scans with the superposition of the corresponding muscles’ manual segmentation and indications of the muscles’ names. **A** Thigh case; **B** Leg case

We processed the input volume with a slice-wise approach. Figure 1 shows muscle segmentation of an exemplary subject, with segmented muscles reported in the figure legend.

Each manual segmentation map was preprocessed through the application of consecutive area opening and closing filters, with an area threshold of 4 pixels, to eliminate small structures which resulted from noise in the registration operation of the MESE onto the GRE dataset.

We applied data augmentation to the available annotated slices in the training and validation datasets to gain robustness in the network predictions on unseen cases and to make the network learn realistic deformations without these being represented in the available training data. In particular, on each training and validation image and on each corresponding manual segmentation we randomly applied elements in the following sequence of transformations (bicubic spline interpolation was used for the input images, and nearest-neighbor interpolation was used for the binary segmentation masks):

- *Horizontal and vertical translations:* separate independent translations of the left and right thighs (or legs) per image in the horizontal and vertical directions, with bi-cubic spline interpolation. The amounts of each translation were uniformly sampled in an interval of values computed per image with the maximum value given by the shortest distance of the thighs (or legs) to the image borders. These transformations enhanced invariance with respect to the relative position between the left and right thighs (or legs) in the training process;
- *Rotations:* independent rotations of the left and right thighs (or legs) by amounts uniformly sampled per image in the interval $[-7,7]$ degrees, with bi-cubic spline interpolation. These transformations enhanced rotation-invariance in the training process;
- *Piecewise affine transformations:* separate affine transformations applied on each neighborhood of points on a $4\times 4$ grid, with each grid point moving of an amount sampled from a normal distribution with scaled amplitude randomly sampled per image from the interval [0.1, 1] percent of the image height/width. Bi-cubic spline interpolation was chosen to determine per–pixel values for the transformations. These transformations enhanced local distortions-invariance in the training process;
- *Elastic transformations:* local transformations obtained in terms of displacement fields with Gaussian kernel smoothing, with strength uniformly sampled per image from the interval [0, 20] and standard deviation of the kernel uniformly sampled per image from the interval [5, 10]. Bi-cubic spline interpolation was chosen to determine new pixel values for the transformations. These transformations enhanced elastic distortions-invariance, representing realistic tissue variations, in the training process.

The aforementioned data augmentation was applied to the available dataset of 770 thigh and 330 leg slices to obtain 5000 annotated images for thigh and leg respectively. We randomly separated this augmented dataset into a training dataset of 4500 elements and a validation dataset of 500 elements, to perform a cross-validation analysis on the network performance.

### Deep learning analysis

**Fig. 2:**
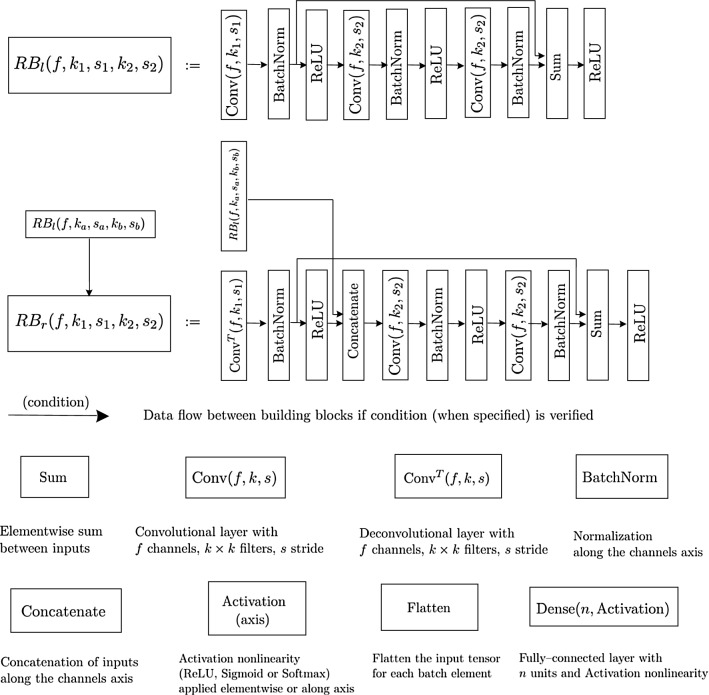
Building blocks of the networks’ architectures, with descriptions of the performed operations

We considered the segmentation problems for the thigh’s and leg’s muscles as multi-class localized classification problems for the 2D images with 13 and 7 classes (comprising background and muscles) respectively, where a class label is assigned to each pixel. We achieved this goal using properly designed deep convolutional neural networks, inserted in a tree-like structure with two branches, where the inner node performs a global classification of the given input 2D image into a thigh’s or leg’s geometry, and according to the classification result two leaf nodes perform the corresponding segmentation task on the same input image. The deep convolutional networks used in this work were customized versions of the *VNet* [20] and *ResNet* [12] architectures, where a contracting network topology is used for the purpose of classification tasks and deep features extraction from increasingly compressed levels of resolution, whereas an expanding network topology is used for resolution decompression and for the segmentation task. The VNet [20] and ResNet [12] architectures were developed to solve problems in biomedical image segmentation and image classification respectively, based on a fully convolutional architecture with the key extension that each convolutional layer learns a residual function. In particular, the VNet architecture was proven to ensure faster convergence during the learning process, mitigating the accuracy degradation with increased network depth, with respect to similar encoder–decoder architectures without residual units (e.g. the Unet network [27]). These networks and their variants have been applied with success in recent years in solving different image segmentation, classification and reconstruction problems [18], becoming the gold–standard DL tools for solving these tasks. The platform nn–Unet [14] recently showed that a basic U-Net, properly calibrated on specific datasets, was able to obtain the highest accuracy over quite different biomedical semantic segmentation tasks with respect to other even more sophisticated architectures. We thus choose to use VNet and ResNet architectures in our work, properly calibrated on our dataset (as will be explained in the sequel). Since we run our DL implementation on a CPU, we choose to use residual units to possibly accelerate the convergence of the training process and limit the needed computational resources. Before going into the details and rationale of the networks, we report in Fig. 2A graphical representation of the building blocks of the networks. The network weights were initialized from a Glorot normal distribution [11], and batch normalization [13] was applied at different levels, which normalized the distributions of the layers input and helped in quickening the learning convergence for deep networks. Each convolution and deconvolution operation was applied with appropriate constant padding, to keep equal dimensions between its input and output. The architecture of the residual block (RB) layers $RB_l$ and $RB_r$ was based on the scheme *Convolution–Batch Normalization–Skip Connection–Activation*, which proved to give optimal convergence properties between the different ResNet implementations analyzed in literature. The network architectures and the learning algorithms were implemented in the *Tensorflow* platform [1], using the deep learning interfaces provided by the *Keras* API [8]. The resulting DNN automatic segmentation tool has been shared as an opensource repository, available at [2].

### Network architecture

**Fig. 3:**
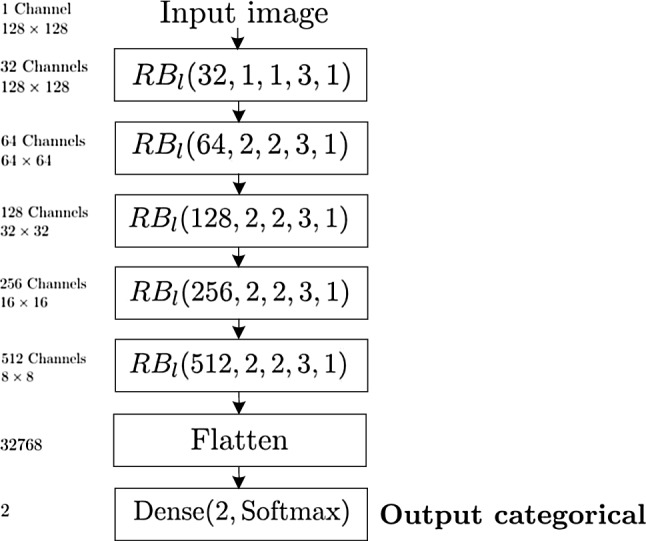
Graphical representation of the network’s architecture for the classification task. The number of channels, spatial dimensions and number of neurons are indicated next to each building block, together with the indications of the input and the output of the data flow

**Classification Network.** In Fig. 3 we report the network’s architecture for the classification task into the thigh’s or leg’s category.

The classification network takes as inputs down-sized images (to $128 \times 128$ size) through cubic spline interpolation and anti–aliasing, to reduce the computational burden. The input image goes through 5 residual block layers $\text {RB}_l$ with doubled channel dimension and halved resolution at each level, extracting features at different spatial aggregation levels (receptive fields). The first residual block applies a first convolutional layer with 32 channels and unit kernel filter and stride, to map the input image to a first set of normalized outputs as a shortcut, after batch normalization, for the residual map. Then, a sequence of 2 convolutional layers with the same channel dimension, a $3\times 3$ kernel filter and a unit stride are applied, to extract independent translation-invariant features at this receptive field resolution after the application of nonlinear units. The remaining four residual blocks apply a first convolutional layer with doubled channel dimension with respect to the previous layer and a kernel filter and stride of dimension 2, working both as a down-sampling and as a shortcut, after batch normalization, for the corresponding residual map. Then, a sequence of 2 convolutional layers with the same channel dimension, a $3\times 3$ kernel filter and a unit stride is applied. Finally, all the extracted features at the different depth levels are collected into a vector of output neurons and used as an input to a fully connected layer for the binary classification task. The output of this final layer (indicated as **Output categorical** in Fig. 5) consists of a two-dimensional vector of probabilities to belong to a specific category, given the one-hot representation (1, 0) for the thigh class and (0, 1) for the leg class.

**Fig. 4:**
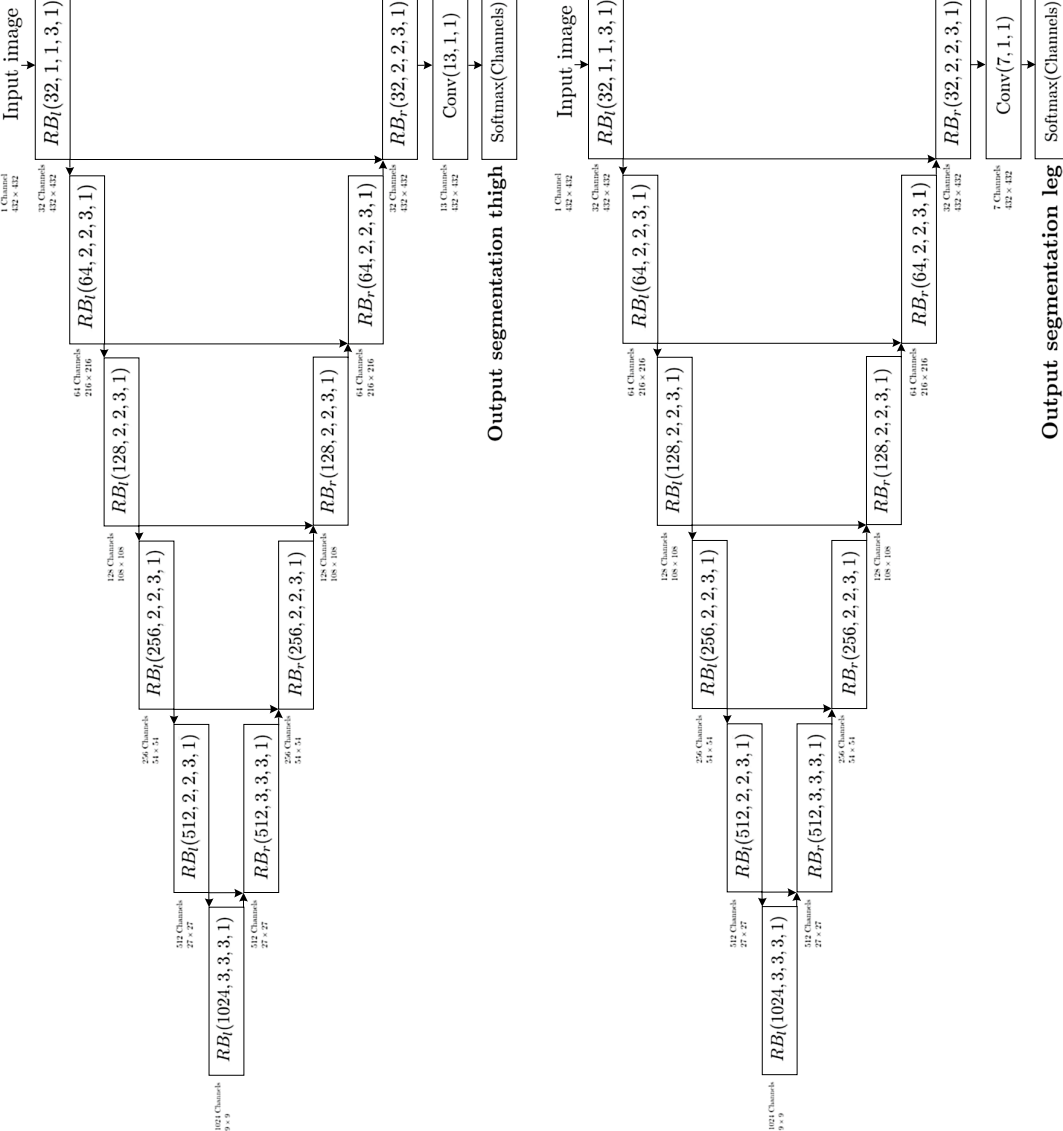
Graphical representation of the network’s architecture for the segmentation tasks. The number of channels, spatial dimensions and number of neurons are indicated next to each building block, together with the indications of the input and the different outputs of the data flow

**Segmentation Networks.** Figure 4 graphically represents the network’s architecture for the segmentation of both thigh’s and leg’s MRI.

The segmentation networks take as inputs the images with full $432\times 432$ size. They are customized versions of the *VNet* [20], consisting of a fully convolutional architecture with a contracting path, composed by a sequence of 6 residual blocks $RB_l$, and an expanding path, composed by a sequence of 6 residual blocks $RB_r$. The first 5 residual blocks of the contracting path apply the same operations as the 5 residual blocks of the classification network. To extend the receptive field to cover the spatial resolution of the full $432\times 432$ images and to introduce a higher number of features at more abstract aggregation levels, which is necessary to solve the segmentation task, we add a sixth layer with 1024 channels and a 1/3 downsampling. The 6 residual blocks of the expanding path increase the spatial resolution in a symmetric way with respect to the contracting path, halving the channel dimension at each level and concatenating with the corresponding resolution features from the contracting path to recover context information. A final convolutional layer with unit kernel filter and stride is applied to map the 32 channels space to the desired probabilistic space with dimension given by the proper number of classes, after the application of the $\text {Softmax}$ nonlinearity pixelwise.

**Fig. 5:**
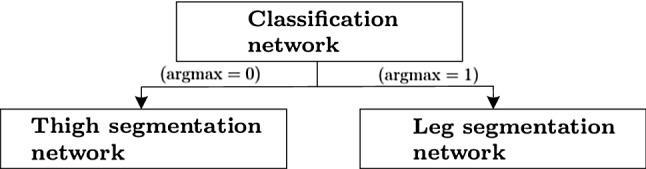
Graphical representation of the network’s architecture as a tree-like structure

We note that the use of small kernel filters (up to dimension $3\times 3$) gave us the possibility to go deeper into the network’s architecture, introducing a fewer number of weights with respect to bigger filters when covering the same receptive fields, at the expense of adding additional layers. This reduced the dimensionality of the network and the computational burden. Also, it introduced smooth variations in the receptive fields between the different layers, distributing the spatial resolution of the extracted features uniformly on the spatial domain and thus covering the patterns’ variability at each spatial scale. To obtain this result, we had to ensure that the receptive fields cover the whole extension of the greatest objects detectable in the images (such as the whole thigh or leg). In Table 1 we report the progression of the receptive fields for each layer in the classification network (with input $128\times 128$ images) and in the contracting path of the segmentation network (with input $432\times 432$ images) in the tree-like network in Fig. 5.

**Table 1:** Receptive fields associated to each convolutional operation in the successive residual blocks $\text {RB}_l$

	Receptive fields
$\text {RB}_l(32,1,1,3,1)$	$1\times 1$, $3\times 3$, $5\times 5$
$\text {RB}_l(64,2,2,3,1)$	$6\times 6$, $10\times 10$, $14\times 14$
$\text {RB}_l(128,2,2,3,1)$	$16\times 16$, $24\times 24$, $32\times 32$
$\text {RB}_l(256,2,2,3,1)$	$36\times 36$, $52\times 52$, $68\times 68$
$\text {RB}_l(512,2,2,3,1)$	$76\times 76$, $108\times 108$, $140\times 140$
$\text {RB}_l(1024,3,3,3,1)$	$188\times 188$, $284\times 284$, $380\times 380$

We can observe from Table 1 that the receptive fields span uniformly through all the relevant spatial dimensions for $128\times 128$ (first 5 residual blocks, reaching up to dimension $140\times 140$) and for $432\times 432$ images (all six residual blocks). In this latter case, we must consider that a single thigh or leg object extends up to half of the image, and the dimension $380\times 380$ contain information about the single thigh (or leg) and the relative positions between left and right thighs (or legs).

**Networks concatenation.** Figure 5 graphically represents the concatenation of the classification and segmentation networks for the classification and segmentation of both thigh’s and leg’s MRI.

It consists of a tree-like structure, where the inner node performs the classification of the 2D downsized input image into the thigh’s or leg’s category. The two branches of the network perform an $\text {argmax}$ operation on **categorical Output**, splitting the data flow towards the left or right leaves depending on the classification outcome: if $\text {argmax}=0$, the input image with full $432\times 432$ size is processed by the left segmentation network with output a probabilistic segmentation for 13 classes (**Output segmentation thigh**), whereas, if $\text {argmax}=1$, it is processed by the right segmentation network with output a probabilistic segmentation for 7 classes (**Output segmentation leg**).

### Hyperparameters optimization, training and evaluation

The network was trained on the augmented dataset of input images and corresponding manual segmentations by means of a stochastic gradient descent method, including data subsampling in mini-batches and dropout regularization in the input layer. The accuracy of the network was monitored during training both on the training and validation datasets. We used the *AMSGrad* variant of the *Adam* algorithm [15, 25] as an efficient method for stochastic optimization both from the computational and the convergence rate points of view. We also added $L^2$ weights regularization to the objective functions to reduce overfitting.

We first tuned the hyperparameters of the training algorithms by means of the *hyperband* algorithm [17], which adaptively allocate computational resources in an efficient way, choosing as a measure of configurations’ performance the evaluation metrics on the validation dataset and exploring the discrete space of hyperparameters $(\text {lr},\text {dr},\text {reg})\in (0.0001-0.01) \times (0 - 0.5) \times (0 - 0.1)$, for 20 epochs keeping fixed the batch size to 5. Here, $\text {lr}$ is the learning rate, $\text {dr}$ is the dropout rate and $\text {reg}$ is the factor for the $L^2$ weights regularization. Note that, thanks to the algebraic preconditioning introduced by the use of residual maps, the learning rate can take higher values than the typical optimized values given in [15].

After hyperparameters optimization, the training algorithm was implemented with a scheduling which reduced the learning rate of the gradient method by a factor of 1/2 when no improvements in the validation loss were observed after 4 epochs, which helped in overcoming plateau domains and local minima of the loss objective functional.

We chosed the *Categorical Cross–Entropy* $-\sum _{i=1}^{2} \text {gt}_i\log (\text {sf}_i)$, where $\text {gt}_i$ is the ground truth score of class *i* and $\text {sf}_i$ is the output of the *softmax* activation, as the loss function for **Output categorical** in the classification part of the network. We moreover measured the classification network’s accuracy on a given dataset by means of the *Categorical Accuracy* metrics, which is defined as the percentage of predicted values that matches with the ground truth values. For what concerns the segmentation tasks, we considered a class-balanced *weighted Cross–Entropy* both for **Output segmentation thigh** and **Output segmentation leg**. The weights were chosen, as in [27], to compensate the different frequency of pixels belonging to a certain class in the training dataset, thus giving more importance to foreground pixels than background ones during learning, giving in particular the most importance to pixels in small muscles, which are more difficult to segment. Moreover, the background regions separating neighboring muscles, computed using morphological operations as in [27], were associated to large weights in order to force the network to learn separation borders and background regions between muscles. The weighted Cross-Entropy loss function had the following form

1 $$\begin{aligned} L=-\sum _{\mathbf {x}\in \varOmega }w(\mathbf {x})\log (p_{l(\mathbf {x})}(\mathbf {x})), \end{aligned}$$

where $p_{l(\mathbf {x})}$ is the output value of the Softmax layer at the pixel value $\mathbf {x}\in \varOmega$ associated to the pixel’s true label $l(\mathbf {x}) \in \{1,\dots ,13\}$ or $\{1,\dots ,7\}$, and

2 $$\begin{aligned} w(\mathbf {x})=w_{l(\mathbf {x})}(\mathbf {x})+w_0\exp \biggl (-\frac{(d_1(\mathbf {x})+d_2(\mathbf {x}))^2}{2\sigma ^2}\biggr ), \end{aligned}$$

with $w_{l(\mathbf {x})}(\mathbf {x})$ the inverse of the frequency of the true class $l(\mathbf {x})$ in the training dataset and $d_1(\mathbf {x})$ and $d_2(\mathbf {x})$ the distances of pixel $\mathbf {x}$ to the nearest muscle and second nearest muscle respectively. The value of $\sigma$ was chosen to represent the maximum distance between neighboring muscles. We set $w_0=10$, $\sigma =7$ for the thigh dataset and $\sigma =8$ for the leg dataset. Finally, the segmentation’s accuracy was measured by means of the *Dice* coefficient (DSC)

$$\begin{aligned}\text {{DSC}}=\frac{2\text {TP}}{\text {FP}+2\text {TP}+\text {FN}},\end{aligned}$$

which is a standard metrics for the overlap between the manual and the automatic segmentation, where TP, FP and FN are the numbers of true positive, false positive and false negative.

### Network testing with a qualitative assessment for mild and severe disease conditions

We tested the performance of the network on the 10 patients of the test dataset, which were unseen during the learning process, by measuring the DSC between the manual and DNN generated segmentations for both their thighs and legs. As a secondary aim, to qualitatively test the performance of the network in the cases of mild and severe fat infiltrations, the 10 patients of the test dataset were chosen to include 5 subjects with mild and 5 subjects with severe fat replacement, on the basis of visual assessment of SE scans by the Mercuri scale [19].

## Results

**Fig. 6:**
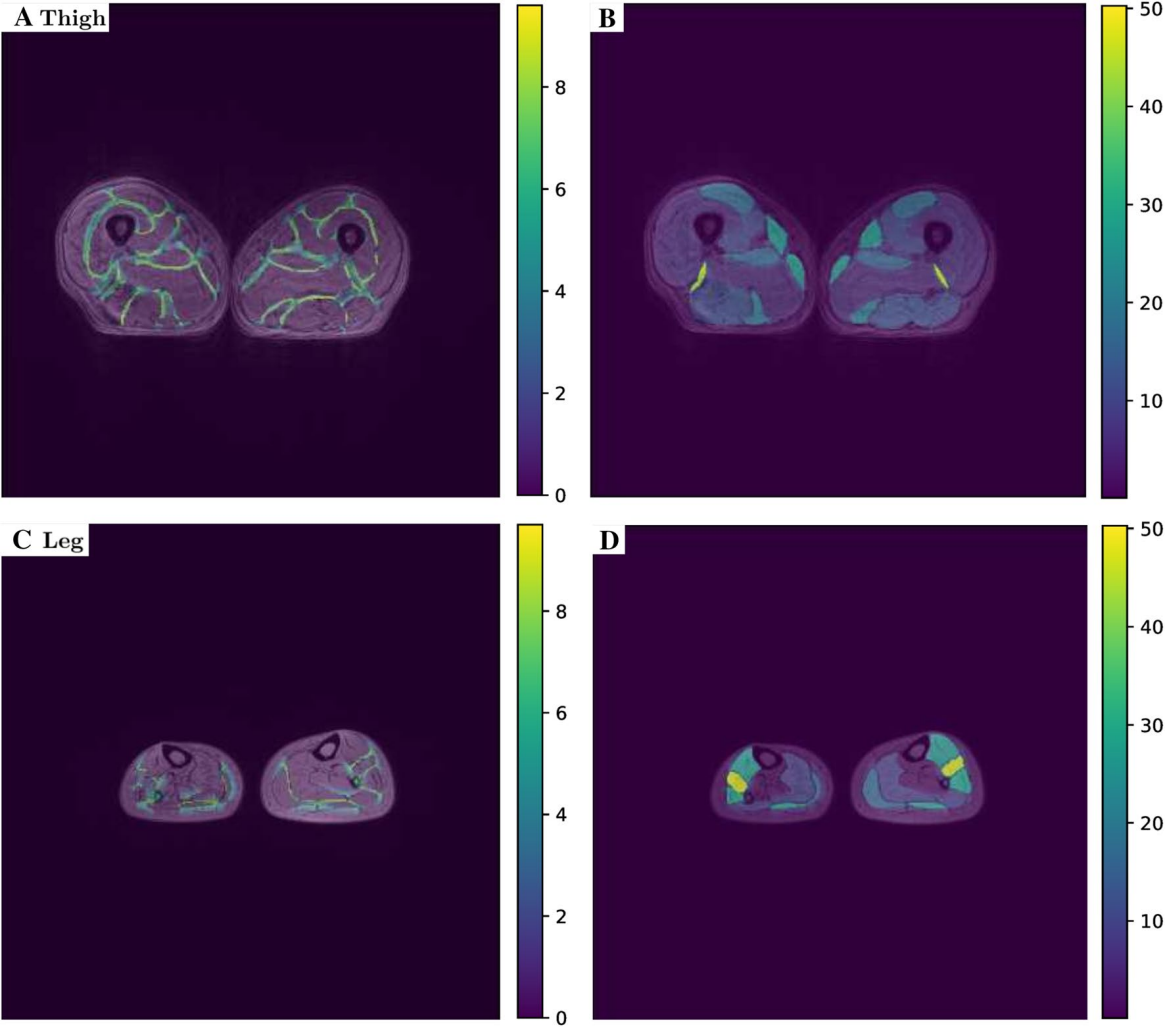
Illustrative example of thigh and leg plots of the weight maps (2). **A** Weights map associated to the background regions separating neighboring muscles for the thigh case; **B** Full weight map for the thigh case; **C** Weights map associated to the background regions separating neighboring muscles for the leg case; **B** Full weight map for the leg case

To illustrate the results of the DNN input creation step, in Fig. 6 we show an illustrative example for the thigh and leg geometries with the plots of the weight maps associated to the background regions separating neighboring muscles [(second term in the right–hand side of (2)] and of the full weight maps (Eq. 2). We can observe that the background regions separating neighboring muscles are associated to a high value of the weight, while the highest weight values are associated with the smallest muscles.

### Hyperparameters optimization, training and validation

We first tuned the hyperparameters of the training algorithm for the left segmentation network in Fig. 5, working on the thigh dataset, and we obtained the optimized values $\text {lr}=0.009765$, $\text {dr}=0.2$, $\text {reg}=0.01$, by which averaged DSC of 0.8744 on the training dataset and 0.8487 on the validation dataset were obtained after 20 epochs. We used these optimized values of the hyperparameters also for the other segmentation and classification networks in the tree.

**Fig. 7:**
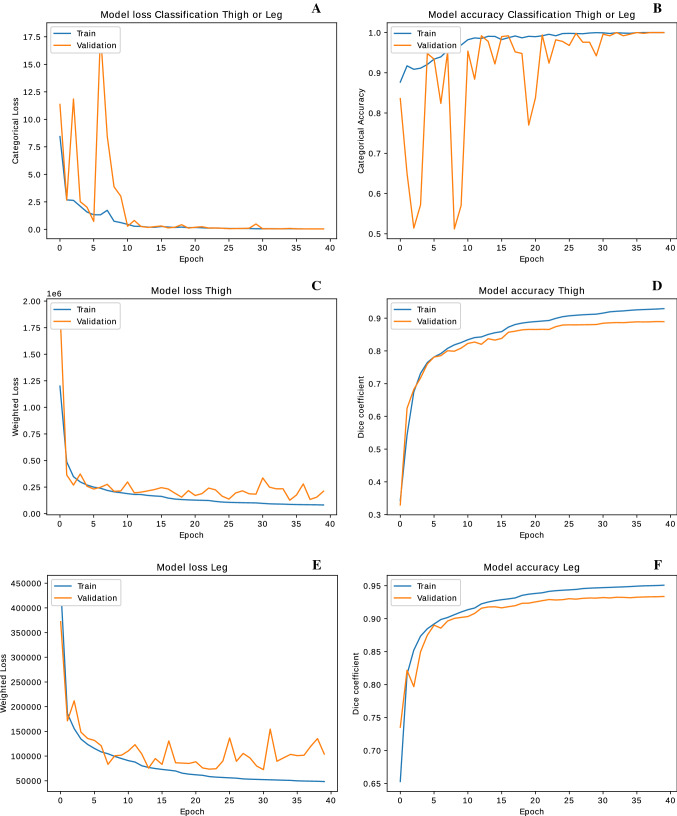
Plots of the model losses and model accuracies during the training of the classification network (**A** and **B**), the thigh segmentation network (**C** and **D**) and the leg segmentation network (**E** and **F**)

In Fig. 7 we show the plots of the model losses and model accuracies during the training, with optimized hyperparameters, of the classification and segmentation network nodes in the tree-like architecture in Fig. 5.

We found that the *Categorical accuracy* of the classification network and the DSC of the segmentation networks had an overall monotonical increase during training on both the training and validation datasets, reaching a plateau which invariably happens after 40 epochs of training for all the networks.

In Table 2 we also report the *Categorical accuracy* and DSC for the classification and the segmentation networks obtained after 40 epochs of training.

**Table 2:** Model accuracy after 40 epochs

	Train accuracy	Validation accuracy
Classification network	Categorical	Categorical
	1.0	1.0
Thigh segmentation network	DSC	DSC
	0.9292	0.8894
Leg segmentation network	DSC	DSC
	0.9507	0.9336

We thus obtained $100 \%$ accuracy of the thigh-leg classification network on both the training and the validation dataset. We highlight the fact that, since the classification network must solve a binary classification problem based on the overall extended spatial features which distinguish between the thigh’s and the leg’s morphology, we found that working on down-sized images to $128\times 128$ dimension was sufficient to achieve $100\%$ accuracy for the classification problem. Indeed, it was unnecessary to extract localized features from the full resolution image to solve this task. We obtained high DSC for both the thigh and leg segmentation networks, namely $93\%$ and $95\%$ respectively on the training dataset, and $89\%$ and $93\%$ respectively on the validation dataset.

**Fig. 8:**
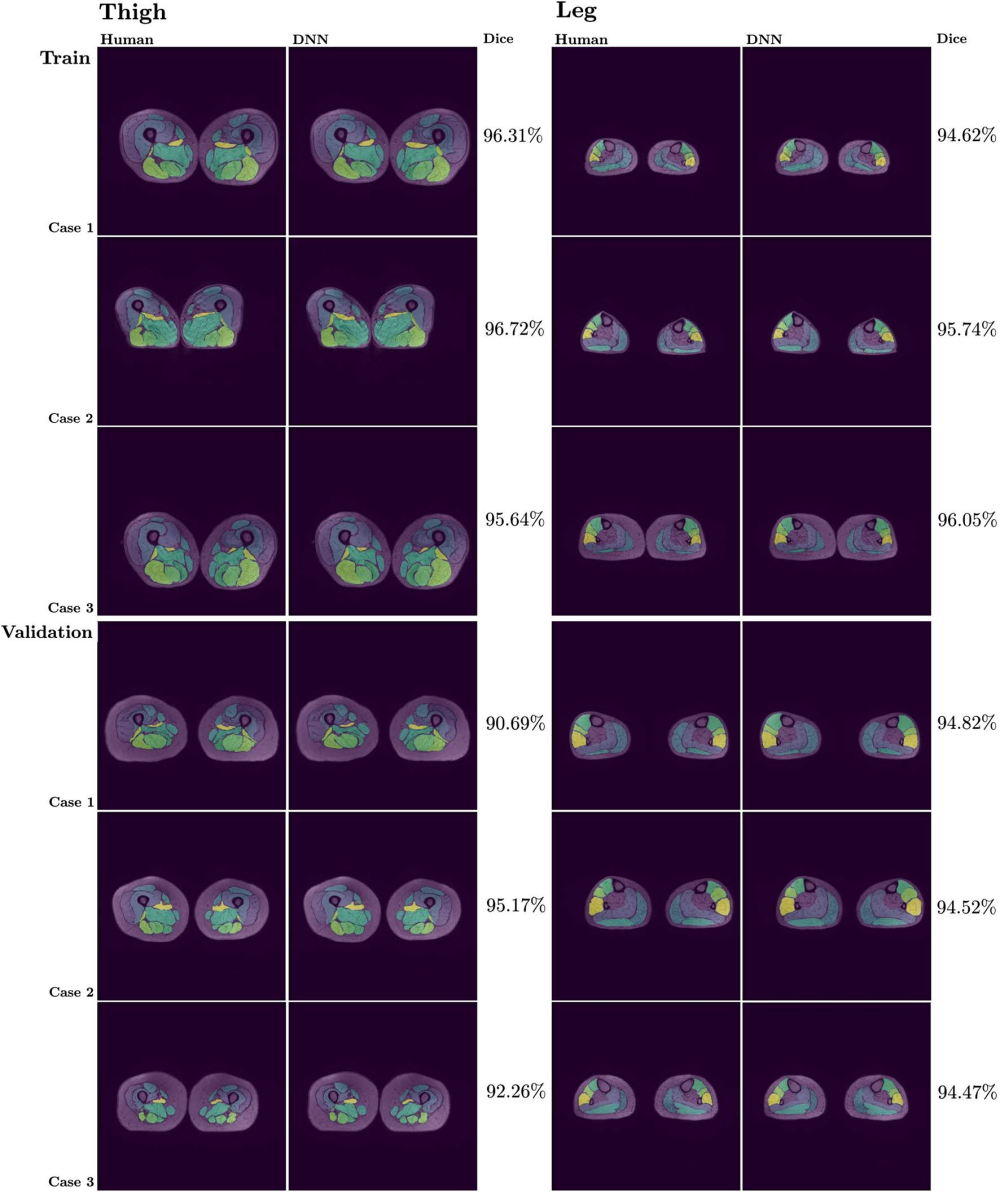
Illustrative comparisons between the manual segmentation and the network (DNN) generated segmentation for three elements in the training and three elements in the validation datasets, for both the thigh and leg case, with the corresponding *Dice coefficient* score

In Fig. 8 we report illustrative comparisons between the manual segmentation and the DNN generated segmentation for three elements randomly chosen in the training dataset and three elements randomly chosen in the validation dataset, for both the thigh and the leg case.

### Network testing

With regards to the test dataset including selected subgroups of subjects with mild or severe fat replacement (see Sect. 2.9), we found that the network segmentation had good and comparable performances for both mild and severe cases, with average $88\%$ and $93\%$ accuracies, respectively for the thigh and the leg, for the subjects with mild fat replacement, and average $87\%$ and $93\%$ accuracies, respectively for the thigh and the leg, for the subjects with severe fat replacement. In Table 3 we report the average DSC over the slices, obtained by the network for the 10 test subjects, separated into two subgroups with mild or severe fat replacement.

**Table 3:** Average DSC for the 10 test subjects, with an indication of their disease severity

	Thigh	Leg
	Average DSC	Average DSC
*Mild*		
Subject 1	0.9009	0.9367
Subject 2	0.9016	0.9310
Subject 3	0.8531	0.9319
Subject 4	0.8651	0.9341
Subject 5	0.8892	0.9243
*Severe*		
Subject 6	0.8762	0.9247
Subject 7	0.8765	0.9295
Subject 8	0.8695	0.9303
Subject 9	0.8923	0.9331
Subject 10	0.8643	0.9285

**Fig. 9:**
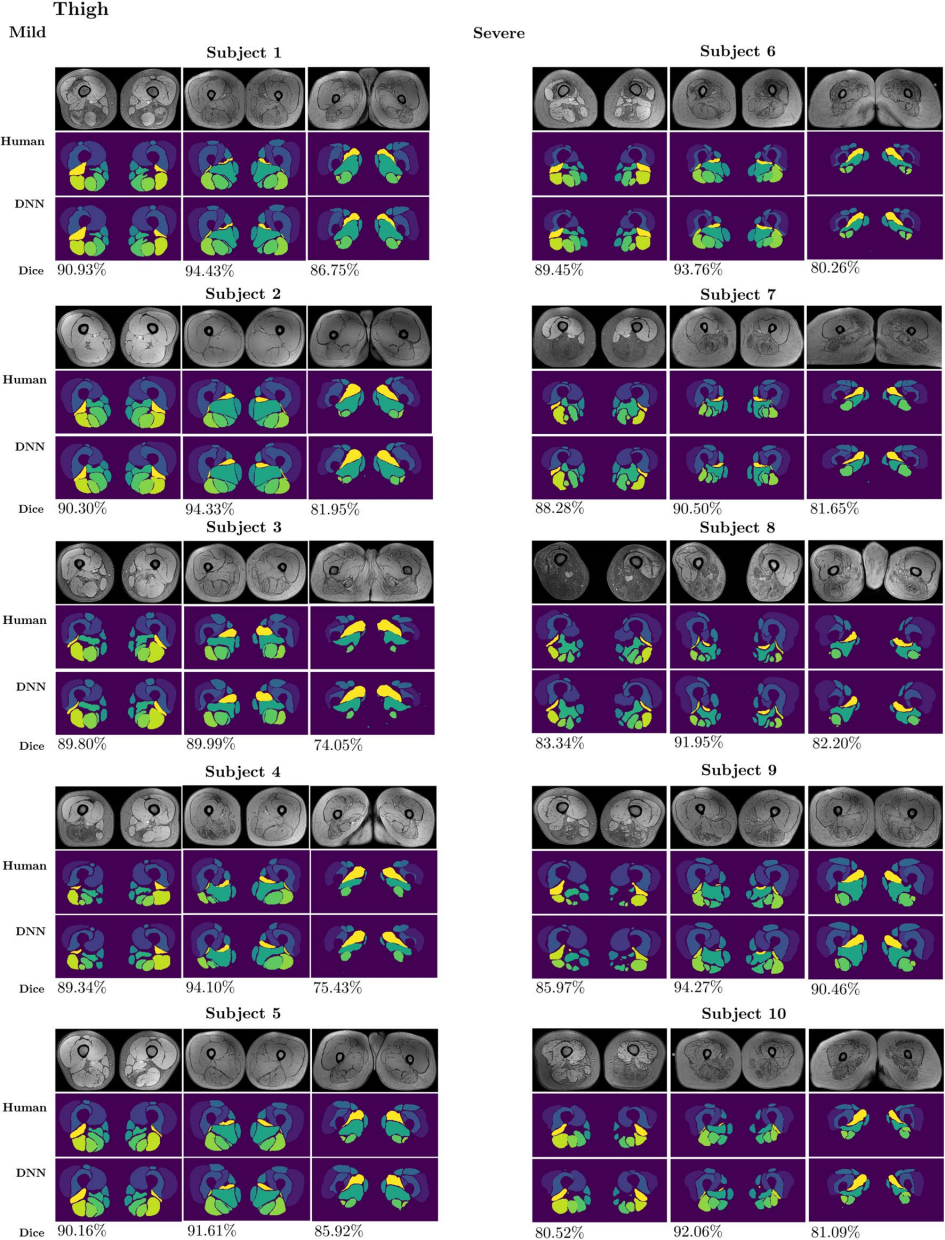
Comparisons between the manual segmentation and the network (DNN) generated segmentation of thigh muscles for 5 patients with mild and 5 patients with severe fat infiltrations in the test dataset. The bottom (leftmost column), inner and top (rightmost column) slices are reported

**Fig. 10:**
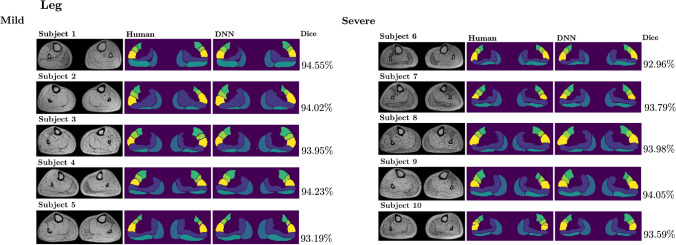
Comparisons between the manual segmentation and the network (DNN) generated segmentation of leg muscles for 5 patients with mild and 5 patients with severe fat infiltrations in the test dataset. The inner slice is reported

Figures 9 and 10 report the 10 selected cases, with an indication of the DSC metrics for single slices. The bottom (leftmost column), inner and top (rightmost column) slices are reported for the thigh, whereas the inner slice is reported for the leg.

**Fig. 11:**
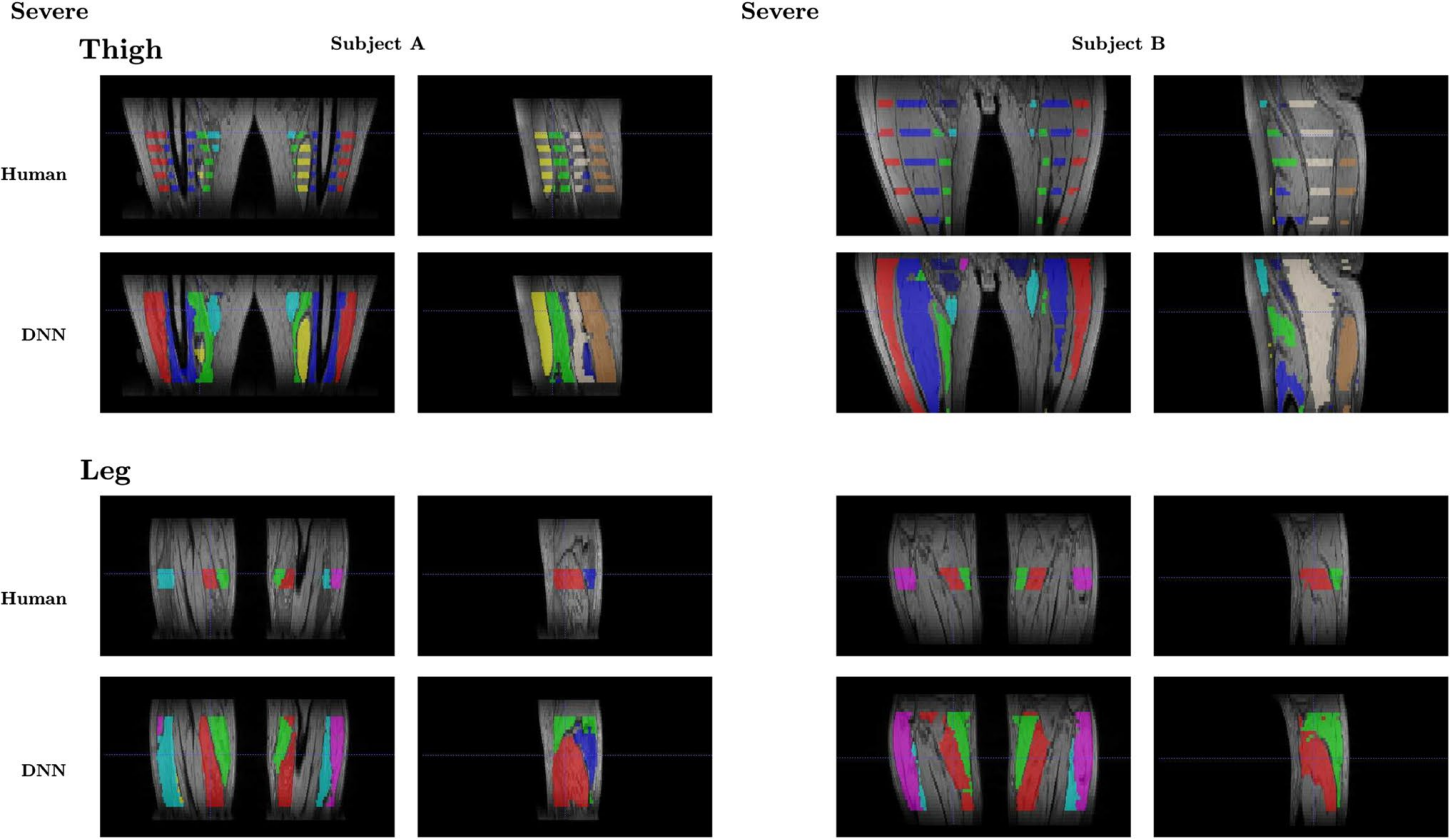
Comparisons between the manual segmentation and the network (DNN) generated segmentation of the thigh and leg muscles for subject A and subject B, shown along with two coronal and sagittal slices

Finally, to evaluate the performance of the DNN on the slices throughout the 3D stack that were unseen during the training process, our expert operator manually segmented 4 additional slices around the middle portion of the thigh and the leg for two subjects randomly chosen in the test dataset, subject A and subject B, both with severe disease involvement (with subject B presenting a higher degree of severity with respect to subject A). In Fig. 11 we show two coronal and sagittal slices along the 3D stack of the thigh and leg images for subject A and subject B, together with the manual and the DNN generated segmentation.

In Table 4 we also report the DSC between the manual and the DNN generated segmentations on the 4 additional slices manually segmented along the 3D stack for both Subject A and Subject B.

**Table 4:** DSC for the 4 additional slices for Subject A and Subject B

	Thigh	Leg
	DSC	DSC
*Subject A*		
Slice 1	0.8041	0.9018
Slice 2	0.8063	0.8954
Slice 3	0.8058	0.9084
Slice 4	0.8113	0.9005
*Subject B*		
Slice 1	0.7514	0.8383
Slice 2	0.7546	0.8529
Slice 3	0.7520	0.8447
Slice 4	0.7327	0.8390

We can observe an overall decrease of the DNN performance to DSC between 0.75 and 0.90 on the slices throughout the 3D stack that were unseen during the training process (at least on the slices around the middle portion of the thigh and the leg of the subjects).

## Discussion

In this study, we approached the automatic segmentation of selected muscles from MRI scans, working on a training dataset composed by thighs and legs of both healthy subjects and patients affected by two different diseases with muscle involvement, and testing the results on a dataset including two subgroups with mild or severe fat replacement. With the aim to standardize and accelerate the process of ROI drawing we developed a deep neural network architecture, consisting of a classifier and two segmentation networks with residual units and contracting and expanding topologies inserted in a tree-like structure, which gave a unified framework for the automatic segmentation of both thigh and leg muscles. Our experience proved the feasibility of a convolutional neural network approach into the automatic segmentation of muscles ROIs for both thighs and legs, with very high accuracy. Specifically, the DNN showed an average DSC of 0.93 and 0.89 on the training and validation sets for the thighs, and of 0.95 and 0.93 on the training and validation sets for the legs, compared to the manually segmented reference ROIs. On the test dataset, an average DSC of 0.88 and 0.87 is found for the thighs of subjects with mild and severe fat replacement respectively, whereas a value of 0.93 is found for the legs of the subjects in both subgroups. We hypothesize that the leg segmentation network we used actually performed better with respect to the thigh segmentation due to a minor variance in the available manually segmented slices along the scanned volume.

The accuracy level obtained by our network was comparable with results obtained by deep neural networks applied to discriminate between different tissues (i.e. fat, muscle tissue, etc.) found in the literature. Such studies which used deep learning methods to discriminate thigh and leg tissues from MRI scans obtained very high accuracy performances, namely DSC of 0.97, 0.94 and 0.80 [4] and 0.96, 0.92 and 0.93 [3] for muscle, fat and inter-muscular adipose tissue respectively. In our study, however, as in [9] we used a different approach as we started from ground truth segmentation of muscles based on their anatomy, resulting in a network capable of replicating the manual segmentation of muscles ROIs done by hand. As muscle MRI studies and also clinical trials often concentrate on single slices or restricted group of muscles as focus for their analysis, the possibility to quantify muscle tissue parameters on a single-muscle level is, in our opinion, of more practical interest. For what concerns tissue segmentation of selected muscles (ROI-based approach), [9] found average DSC values of 0.85–0.93 for the single thigh muscles considered, with the lowest value corresponding to the smallest muscle, while DSC values of 0.78–0.97 have been reported in [26] for small and large muscles respectively. Even if our work exploits 2D slices it reaches results similar to the 3D network topology reported in [26], with the advantage to train only one network for all thigh’s muscles and only one for all leg’s muscles in contrast to [26] in which the authors train individual networks for each target muscles.

As explained in the Methods, the network was trained on the augmented dataset by means of a stochastic gradient descent method, with a schedule of the learning rate to overcome plateau domains of the loss objective functionals. The hyperparameters of the networks were chosen in advance by solving an adaptive optimization problem based on monitoring the DSC on the validation dataset. The proposed supervised training algorithms converged with an overall monotone behavior to a local minimum for each component networks, proving robustness of the learning process. We cross-validated the networks performances on a validation set of unseen slices, which were excluded from the training dataset, and we tested their performances on a test dataset of unseen subjects, obtaining very high DSC values between the human and network generated segmentations, in the order of $90\%$.

In addition to this our classification network obtained a $100\%$ accuracy, both over the training and validation datasets, in classifying between the thigh or leg geometry. This paves the way to a consistent extension of our deep learning network to automatically segment proper labels for different anatomical districts, once the classifier is also trained on a properly adapted dataset from different sequences with different contrast and resolutions.

One secondary aim of our study was to evaluate whether the performance of our DNN was affected by the different level of muscle involvement (i.e more or less fat replaced muscles) in the subjects. We found that when evaluating subjects with mild disease involvement, our DNN showed a high level of accuracy, comparable to that of previous tissue-discriminating networks and also to the previous experience of [9] and [26]. Differently from the reported literature and due to the subjects’ variability in our data set (control subjects and subjects affected by different diseases), a high level of accuracy was also obtained by our DNN when evaluating subjects with the most severe disease involvement.

The current study had some limitations. First, since ground truth segmentations were available only on selected slices of the MRI volume stack (see Sect. 2.3), the DNN performance on the other slices that were unseen during the training process is lower than on selected slices, and manual corrections were needed on the DNN generated segmentations on unseen slices in the overall subject volumes. Second, the DNN was trained and tested only on the available thigh and leg datasets, achieving high accuracy performances, but an external validation and eventually incremental training on independent datasets acquired with different sequence parameters or even different sequences or systems would be further needed to ensure the reproducibility of our segmentation tool to clinical practice. Also no evaluation was performed on data from healthy volunteers. As a future development, incremental learning will be used to incorporate information from other contrasts and thus aim at a higher generalizability of the model.

## Conclusions

In this study, we explored the applicability of deep neural networks in ROI drawing of muscles of the lower limbs, with promising results in terms of accuracy compared to the standard manual reference currently in use. The application of neural networks to substitute or at least greatly accelerate the work of human operators in ROI drawing can be extremely helpful in clinical studies, where a large amount of data have to be analyzed. Once reliable dedicated datasets of muscle ROIs are collected, deep neural networks can be promisingly applied for segmentation of other sequences with different contrast and image resolution and also to different anatomic districts.
